# Supplementary material for: Influence of the Catecholamine Syringe Changeover Method on Patients’ Blood Pressure Variability: A Single-Center Retrospective Study
Source: Nurs Rep. 2025 Sep 23;15(10):345. doi: 10.3390/nursrep15100345 (PMC12567164; doi:10.3390/nursrep15100345)
Supplement: Supplementary file 1 [file nursrep-15-00345-s001.zip › Supplemental Table S4.pdf]

Supplemental Table S4. Analysis Results of Syringe Exchange in Severe Shock (Sub-analysis)

		Parallel exchange (n=19)	Quick exchange (n=26)	P value
Coefficient of variation				
After 5 minutes	SBP	0.024(0.010-0.059)	0.023(0.012-0.044)	0.927
	DBP	0.022(0.012-0.047)	0.023(0.012-0.049)	0.945
	MBP	0.022(0.010-0.056)	0.020(0.010-0.043)	0.646
After 10 minutes	SBP	0.026(0.016-0.065)	0.046(0.029-0.087)	0.168
	DBP	0.043(0.018-0.054)	0.039(0.021-0.052)	0.936
	MBP	0.045(0.017-0.063)	0.038(0.024-0.073)	0.408
After 15 minutes	SBP	0.040(0.018-0.068)	0.049(0.030-0.077)	0.168
	DBP	0.044(0.032-0.072)	0.040(0.025-0.059)	0.629
	MBP	0.046(0.025-0.061)	0.046(0.025-0.069)	0.613
Absolute value of the change				
After 5 minutes	SBP	-1.0(-4.0-2.0)	-2.5(-5.5-1.8)	0.604
	DBP	-1.0(-2.5-1.5)	-0.5(-2.0-2.0)	0.936
	MBP	-1.0(-2.7-1.7)	-1.0(-2.6-1.3)	0.679
After 10 minutes	SBP	-1.0(-2.5-3.0)	-0.5(-4.0-8.0)	0.791
	DBP	0.0(-3.0-1.0)	1.0(-2.0-3.0)	0.510
	MBP	0.3(-3.2-1.1)	0.2(-2.6-4.6)	0.490
After 15 minutes	SBP	-3.0(-4.0--0.5)	-4(-5.0-2.3)	0.729
	DBP	-2.0(-3.0-2.0)	-0.5(-2.0-2.8)	0.235
	MBP	-2.3(-3.3-2.3)	-1.3(-2.9-2.7)	0.434

This table is shown with median (25-75th percentile)

SBP: systolic blood pressure, DBP: diastolic blood pressure, MBP: mean blood pressure
