# Supplementary material for: Influence of the Catecholamine Syringe Changeover Method on Patients’ Blood Pressure Variability: A Single-Center Retrospective Study
Source: Nurs Rep. 2025 Sep 23;15(10):345. doi: 10.3390/nursrep15100345 (PMC12567164; doi:10.3390/nursrep15100345)
Supplement: Supplementary file 1 [file nursrep-15-00345-s001.zip › Supplemental Table S1.pdf]

Supplemental Table S1. The list of exclusions

Reasons for exclusion	Number
Treatments that may affect hemodynamics during the measurement period	291
(a) changes in catecholamine preparation infusion rate	90
(b) initiation or changes in sedative drug infusion	19
(c) administration of antipyretics, diuretics, steroids, or electrolytes	5
(d) bolus fluid administration or blood transfusion	2
(e) initiation of continuous dialysis, circuit exchange, or changes in fluid removal	13
(f) initiation or changes in mechanical circulatory support	9
(g) changes in ventilator settings	1
(h) body positioning or hygiene care	123
(i) airway secretion suction	4
(j) therapeutic interventions	9
(k) rehabilitation	3
(l) movement or transfer within the bed	11
(m) delirium symptoms	2
Cases where invasive arterial pressure measurement was not possible	75
(n) leaving the room for tests	2
(o) arterial line issue	7
(p) missing measurement data	6
(q) blood gas analysis	8
(r) non-insertion of arterial pressure line	52
