# Supplementary material for: Influence of the Catecholamine Syringe Changeover Method on Patients’ Blood Pressure Variability: A Single-Center Retrospective Study
Source: Nurs Rep. 2025 Sep 23;15(10):345. doi: 10.3390/nursrep15100345 (PMC12567164; doi:10.3390/nursrep15100345)
Supplement: Supplementary file 1 [file nursrep-15-00345-s001.zip › Supplemental Table S2.pdf]

Supplemental Table S2. Patient Background After Propensity Score Matching

	Parallel exchange (n=76)	Quick exchange (n=76)	P value
Age	67.5 (64.3-83.3)	68.0 (50.8-81.0)	0.090
Sex（male, %）	49 (64.4)	49 (64.4)	1.000
Primary disease			
Infection（n, %）	36 (47.4)	34 (44.7)	0.745
Post-cardiac arrest（n, %）	16 (21.1)	22 (28.9)	0.261
Burns（n, %）	17 (22.4)	11 (14.4)	0.209
Hypothermia（n, %）	3 (3.9)	2 (2.6)	0.649
Medical history			
Hypertension（n, %）	26 (34.2)	22 (28.9)	0.485
Diabetes（n, %）	17 (22.4)	25 (32.9)	0.147
Heart disease（n, %）	18 (23.7)	23 (30.3)	0.361
Number of catecholamines used (single, %)	32 (42.1)	39 (51.3)	0.255
Total flow rate of the exchange route at the time of exchange (ml/h)	5.3 (3.4-9.1)	5.8 (4.0-10.0)	0.385
Infusion rate of the exchanged drug (ml/h)	4.1 (2.7-6.3)	4.7 (2.8-6.3)	0.465
Dosage of the exchanged drug (γ)	0.2 (0.1-0.2)	0.2 (0.1-0.3)	0.932
The coefficient of variation in mean blood pressure during the 30 minutes before syringe exchange	0.037 (0.023 – 0.054)	0.033 (0.019 – 0.052)	0.488
The time of exchange（Night, %）	44 (57.8)	36 (47.3)	0.194

This table is shown with n (%) or median (25-75th percentile)
