# Supplementary material for: Influence of the Catecholamine Syringe Changeover Method on Patients’ Blood Pressure Variability: A Single-Center Retrospective Study
Source: Nurs Rep. 2025 Sep 23;15(10):345. doi: 10.3390/nursrep15100345 (PMC12567164; doi:10.3390/nursrep15100345)
Supplement: Supplementary file 1 [file nursrep-15-00345-s001.zip › Supplemental Table S3.pdf]

Supplemental Table S3. Patient Background in Sub-analysis Limited to Syringe Exchange in Severe Shock

	Parallel exchange (n=19)	Quick exchange (n=26)	P value
Age	67.0 (57.0-79.0)	68.0 (51.0-73.0)	
Sex（male, %）	14 (73.7)	14 (53.8)	0.148
Primary disease			
Infection（n, %）	11 (57.9)	7.0(26.9)	0.037
Post-cardiac arrest（n, %）	3 (15.8)	9 (34.6)	0.142
Burns（n, %）	0 (0.0)	7 (26.9)	0.014
Hypothermia（n, %）	4 (21.1)	2 (7.7)	0.195
Medical history			
Hypertension（n, %）	4 (21.1)	8 (30.8)	0.393
Diabetes（n, %）	0 (0.0)	3 (11.5)	0.196
Heart disease（n, %）	1 (5.3)	8 (30.8)	0.044
Type of exchanged drug			0.539
Noradrenaline（n, %）	15 (78.9)	20 (76.9)	
Dobutamine（n, %）	4 (21.1)	3 (11.5)	
Vasopressin（n, %）	0 (0.0)	2 (7.7)	
Adrenaline（n, %）	1 (5.3)	1 (3.8)	
Dopamine（n, %）	0 (0.0)	0 (0.0)	
Milrinone（n, %）	0 (0.0)	0 (0.0)	
Normal saline solution to boost catecholamine（n, %）	0 (0.0)	0 (0.0)	
Number of catecholamines used (single, %)	12 (63.2)	18 (69.2)	0.455
Total flow rate of the exchange route at the time of exchange (ml/h)	10.2 (6.8-15.3)	7.5 (6.0-10.5)	0.164
Infusion rate of the exchanged drug (ml/h)	6.0 (5.4-8.6)	5.4 (4.0-6.7)	0.401
Dosage of the exchanged drug (γ)	0.3 (0.2-0.4)	0.3 (0.2-0.5)	0.749
The coefficient of variation in mean blood pressure during the 30 minutes before syringe exchange	0.034 (0.018 - 0.055)	0.043 (0.030 - 0.059)	0.290
The time of exchange（Night, %）	11 (57.9)	12 (46.2)	0.317

This table is shown with n (%) or median (25-75th percentile)
